# Centenarian Studies: Important Contributors to Our Understanding of the Aging Process and Longevity

**DOI:** 10.1155/2010/484529

**Published:** 2011-07-13

**Authors:** Donald Craig Willcox, Bradley J. Willcox, Leonard W. Poon

**Affiliations:** ^1^Okinawa International University, Okinawa 901-2701, Japan; ^2^Okinawa Research Center for Longevity Science, Okinawa 901-2114, Japan; ^3^Pacific Health Research and Education Institute, Honolulu, HI 96813, USA; ^4^Department of Research, Kuakini Medical Center, Honolulu, HI 96817, USA; ^5^Department of Research, Planning and Development, The Queen's Medical Center, Honolulu, HI 96813, USA; ^6^Department of Geriatric Medicine, John A. Burns School of Medicine, University of Hawaii, HI 96817, USA; ^7^The Institute of Gerontology, University of Georgia, UGA, Athens, GA 30602, USA

In the course of the next 10 years, say the watchers of consumer trends, a new generation—Generation C—will emerge. Born after 1990, they are referred to as “digital natives,” now beginning to attend university and enter the workforce, they are expected to transform the world as we know it [1]. The “C” stands for “connected,” “communicating,” “content-centric,” “creative,” and “change”; however, it may just as well stand for “centenarian” as for the first time in history many of this birth cohort will live 100 years or more. The fact is that people are living longer and generally healthier lives than ever before. Mortality, and by some measures incident morbidity and disability, is being delayed considerably in today's elderly [2]. As a result, centenarians, once considered rare, are now becoming commonplace. Indeed, they are the fastest growing demographic group of the world's population, their numbers having roughly doubled every decade since 1950, and they are globally projected to more than quintuple between 2005 and 2030 [3]. 

According to some estimates [3], the odds of living to one hundred have risen from approximately 1 in 20 million to 1 in 50 for women in some low-mortality nations. If progress in reducing mortality continues at the same pace as it has over the past two centuries, which is still being debated [2, 4, 5], many, if not most, children born today in low-mortality countries can expect to become centenarians [6]. The US Social Security Administration [7] forecasts the number of centenarians in the US to surpass one million before the end of this century. Generation C will indeed be in full bloom. 

This unprecedented phenomenon is largely the result of significant public health advancements that markedly reduced early life mortality, principally due to interventions that reduced infectious diseases in the first decades of the 20th century [4]. Lasting effects of such interventions from less lifetime inflammation, and other ancillary benefits, may have contributed [8]. More recently we have seen marked reductions in morbidity and mortality at older ages [9]. Less well understood, these late life health improvements coincide with the aging of younger cohorts who practiced healthier behaviors and had access to better medical care [10]. The culmination of these advances has resulted in the most rapid health improvement in the history of humanity. Centenarians, once rare, are now living testaments to these remarkable health advancements [11]. 

Despite the mounting weight of scientific evidence for the impending appearance of a new generation of oldest old and, moreover, one that might rival Generation X, Y or the baby boomers in social significance, the global implications of this phenomenon have yet to be fully appreciated. Nor have funding agencies in most nations, until recently, realized the value of investing research resources on the study of the oldest old [12]. In fact, the older population (including “young-old” aged 65–75 years) was formerly systematically excluded from clinical trials [13]. Indeed, many of the rare and valuable prospective cohort studies on older adults that exist today were not begun as studies of aging. Instead, younger cohorts were followed for decades for other phenotypes, such as cardiovascular diseases, when prescient researchers and funding bodies began to add aging-related variables, phenotypes, and outcomes. Two such examples are the Honolulu Heart Program [14] and the Framingham Heart Study [15], each with over four decades of extensive followup and several ancillary studies of aging.

Since centenarian studies, like most centenarians themselves, have been a phenomenon of only the past several decades, there has been no large repository of prior biological, psychosocial, demographic, genetic, or clinical data from which to inform researchers, policy makers, or clinicians. Fortunately, this situation has begun to change in recent years, and centenarian studies, once in their infancy, are now themselves beginning to mature. Indeed, the world's longest continuously running centenarian study, the Okinawa Centenarian Study, began in 1975 and is now entering its fourth decade. The longest running centenarian study in the USA, the Georgia Centenarian Study [16], recently celebrated its 20th anniversary by hosting a conference with different centenarian research teams from around the world [17]. Representatives from the Ashkenazi Jewish, Chinese, Danish, French, Georgia, Hawaii, Korean, New England, Okinawa, and Tokyo centenarian studies, as well as the NIA Longevity Consortium, gathered to share information at this conference, many of whom also made contributions to the current special issue. Other major studies have been ongoing for a considerable period of time in Italy, Sweden, Germany, and other areas of the world. 

There is no doubt that centenarian studies are quickly maturing with over a dozen major studies now operational worldwide for a decade (or more) [18] and several more either recently begun or in the planning stages. Therefore, it is timely that the manuscripts in this special issue exemplify the progress being made in this field. 

Past work from centenarian studies has illuminated the field of aging with important discoveries. A brief and necessarily imperfect survey of some such highlights includes the key area of genetics of aging and longevity, where the first so-called “longevity-associated genes” emerged in the 1980s from the study of HLA polymorphisms in Okinawans [19]. Several years later, in the mid-1990s, APOE emerged [20] and was later widely replicated (for review see [21, 22]). Subsequently, there appeared genome-wide association studies (GWAS) as we ushered in the 2000s. As yet, there has been little replication of early GWAS findings [23, 24]. This may be due, in part, to statistical limitations of GWAS studies that require substantially larger sample sizes of the very old for adequate power. This has led to ever larger consortia for meta-analyses and related studies [25–27].

More recent advances in genotyping methodology have allowed for sophisticated, rapid, and inexpensive SNP genotyping, targeted DNA sequencing, and large-scale “deep” sequencing, particularly around “hot” genomic areas. Such developments are helping facilitate rapid discovery as well as rapid replication studies. For example, the original discovery that the evolutionarily conserved FOXO3A gene is important to healthy aging and longevity in humans was found in long-lived Japanese-Americans in Hawaii [28] and within a year was replicated in German and French [29], and Italian [30] centenarian populations as well as three other independent cohorts of oldest old [31]. Multiple other replications followed within 2 years [22]. Other promising gene variants, such as those within the CETP gene, may be population specific since to date robust findings have only appeared in Ashkenazi Jews [32] and Japanese-Americans [33] and they involved completely different gene variants that were either very rare or did not exist in the other population—but have broadly similar biological effects. There will likely be other such discoveries inspired by evolutionarily conserved biological pathways from model organisms of aging [34]. “Epigenetics,” various “omics,” and “mimetics,” much of it inspired by model organism research, is also rapidly coming to studies of aging humans, and centenarian studies will be at the forefront of this new research tide [35].

A small sample of other important findings includes the discovery that centenarian families also seem to be longer-lived and healthier than the rest of us [36]. Centenarians appear to have brothers and sisters, as well as children, who tend to live longer with lower risk for age-associated disease [37, 38]. Population-based studies have revealed that centenarians are overwhelmingly female except in rare areas of the world [39]. Yet despite their superiority in numbers, phenotypic characterization has revealed that the few males who do live to 100 tend to have higher levels of functioning when compared to their female counterparts. Exploring the centenarian phenotype has been of great interest, and understanding aging-related phenotypes has taken on new importance for the gerontological research agenda ([40–42]). Multiple studies (we introduce a few examples and concentrate on review papers) of centenarians have helped quantify and characterize the phenotype of exceptional survivors in terms of aging biomarkers, biochemistry, nutritional status and anthropometry [43–47], inflammation [48–50], cardiovascular risk profiles [51], physical and cognitive functioning [52–57], morbidity profiles [58], personality traits [59], among other aging-related phenotypes [42, 60]. Psychosocial studies have shown that adaptation to the challenges of aging is also a key protective factor for healthy aging and longevity (see a review of key findings in this special issue, by L. W. Poon et al., (2010). This is a mere sprinkling of findings from the numerous manuscripts now available from centenarian studies. This important body of work has helped shape the gerontological research knowledge base and has set a wider agenda for aging research. 

The current special issue helps build on this knowledge base and begins with two important manuscripts that have focused upon the demographic characteristics of this new emerging generation of centenarians. The first manuscript, by R. D. Young et al. (2010) begins by challenging commonly held ideas (both by the lay public and by researchers) regarding exceptional longevity and builds preliminary typologies of extreme longevity myths based upon both field experience investigating claims to extraordinary longevity and data analysis of American Social Security Death Index files of supercentenarians (aged 110 and over). The conclusions are sobering. Despite extraordinary claims to exceptional longevity regularly surfacing in the media and even in respected scientific journals, the majority of age claims over the age of 110 years, and nearly all over the age of 115 years, have turned out to be false. Acceptance of such extraordinary ages without adequate skepticism and evaluation (age validation) undermines responsible scientific research, journalism, and public knowledge in this field. The second manuscript in the issue, by J. Robine et al. (2010), aims to specify the level of mortality selection among centenarians from 5 low-mortality countries (Denmark, France, Japan, Switzerland and Sweden) all part of the 5-Country Oldest Old Project (5-COOP). Three levels of mortality selection were discovered: a milder level in Japan, a stronger level in Denmark and Sweden, and an intermediate level in France and Switzerland. These diverging trends offer an opportunity to study the existence of a trade-off between the levels of mortality selection and the functional heath status of the oldest old in low-mortality countries.

The next two manuscripts in the special issue deal with predictors and dynamic determinants of healthy aging and longevity. J. Arnold et al. (2010) employed morbidity profiles (originally developed by [58]) for their population-based sample from the Georgia Centenarian Study, to determine proportions of centenarians reaching 100 years as survivors (43%), delayers (36%), or escapers (32%) of chronic, age-associated diseases. Diseases fell into two morbidity clusters, one that involves diseases such as CVD, cancer, anemia, and osteoporosis and another associated with dementia. Major barriers to reaching centenarian status in a “healthy state” come from several incident chronic age-related diseases—increasing cancer risk from their sixties, cardiovascular risk from their seventies, and dementia risk from their eighties. Interestingly, 43% of centenarians in this population-based study managed to *escape* a clinical diagnosis of dementia, and, in concert with other studies of the oldest old, few had suffered from cancer. Consistent with their model of developmental adaptation, distal life events contributed to predicting survivorship outcome. Morbidity classification and health status appeared as critical adaptation variables in very late life. A. I. Yashin et al. (2010) in their manuscript on dynamic determinants of longevity, utilize data from the Framingham Study to assess longitudinal changes in physiological indices such as BMI, diastolic blood pressure, pulse pressure, pulse rate, blood glucose, hematocrit, and serum cholesterol. Their primary aim was to investigate the possibility that dynamic properties of age trajectories of these physiological indices could be important contributors to morbidity and mortality. The authors showed that indeed the *rate* of change in physiological state between forty and sixty years served as a good predictor of morbidity and mortality risk later in life and that the rates of decline after reaching the maximum, the actual maximal value itself, and the age at which maximal values were reached were important predictors of morbidity and mortality risk. 

The next two manuscripts of the special issue focus upon assessment of physical capabilities of middle-aged adults, older adults, and the exceptionally old (centenarians). C. D. Ceria-Ulep et al. (2010) investigate the reliability and correlations with age of the balance components of the EPESE and NHANES tests and the Good Balance Platform System (GBPS) in a normal population of adults. It was found that the EPESE and NHANES batteries of tests were not sufficiently challenging to allow successful discrimination among subjects in good health, even older subjects. The GBPS allowed objective quantitative measurements but had low reliability coefficients except for the most difficult testing conditions. Both height and body fat were associated with GBPS scores necessitating adjusting for these variables if using balance as a predictor of future health, particularly in a population witnessing ever-increasing obesity. Assessing physical performance in middle-aged and older adults may be challenging but the broad variation in physical abilities (from independence to bed-bound immobility) found in centenarians makes it extremely difficult to evaluate function using a single instrument. A. E. Cress et al. (2010) utilize data from a population-based sample of 244 centenarians and 80 octogenarians to provide norms on the Short Physical Performance Battery and extend the range of this scale using performance on additional tasks and item response theory (IRT) models, reporting information on concurrent and predictive validity of this approach. Using the original SPPB scoring criteria, 73% of centenarian men and 86% of centenarian women were identified as severely impaired by the scale's original classification scheme. Results suggest that conventional norms for older adults need substantial revision for exceptionally old persons, such as near centenarians and centenarians, and that item response theory methods can be helpful to address floor and ceiling effects found with any single measure.

The next three manuscripts in the special issue deal with biological phenotypes of centenarians. What do centenarians look like underneath the skin? A. von Gunten et al. (2010), in a review article, explore brain aging in the oldest old revealing that pathological substrates of cognitive deterioration, such as the patterns of lesion distribution and neuronal loss, seem to be different in the oldest old compared to those observed in the younger old. In contrast to younger ages where dementia is mainly related to severe neurofibrillary tangle (NFT) formation within adjacent components of the medial and inferior aspects of the temporal cortex, the oldest old tend to display a preferential involvement of the anterior part of the CA1 field of the hippocampus, whereas the inferior temporal and frontal association areas are relatively spared. The authors suggest that microvascular parameters such as mean capillary diameters may be key factors to consider for the prediction of cognitive decline in the oldest old. 

In the next manuscript, M. Suzuki et al. (2010) investigated blood lipid peroxidation and the role of tocopherols in oxidative stress and longevity among Okinawan centenarians. Oxidative stress, inflammation, and aging are intimately linked biological processes that are partly mediated by nutritional status. Finding micronutrients or other natural compounds that might protect against age-related diseases or might slow aging itself is of great interest. Suzuki and colleagues' finding of low plasma levels of lipid peroxides in centenarians compared to younger controls argues for protection against oxidative stress in the centenarian population and is consistent with predictions of the Free Radical Theory of Aging. However, the study did not strongly support a role for vitamin E in this phenomenon. One intracellular tocopherol subtype (beta) was found to be significantly higher in centenarians and may deserve further study. In the next manuscript, C. S. Kwak et al. (2010) do interesting detective work to unravel the mystery of vitamin B12 status in Korean centenarians. They ask how it is possible for Korean centenarians, who ate minimal animal products over the course of their lives, to do relatively well in terms of avoiding vitamin B12 deficiency, a common problem for older people everywhere. The researchers discovered that the traditional plant-based Korean diet consumed by the centenarians was actually providing them with a considerable amount of this important nutrient and, upon screening, found fermented soybean foods such as kimchee and seaweeds to be potent sources of vitamin B12.

Finally, the last two manuscripts in this special issue focus upon the much neglected role of psychosocial factors in reaching centenarian status. Y. Zeng et al. (2010), using a unique data set from the Chinese Longitudinal Healthy Longevity Survey and the largest sample to date of centenarians, show that Chinese centenarians, as reflected in survey response items emphasizing coping and adjustment (such as personal tenacity, optimism, coping with negative moods, secure relationships, and self-control), are significantly more resilient than younger elders in their 90s, 80s or 70s. Their results argue for policies and programs that might promote this characteristic. The last manuscript in the special issue, by L. W. Poon et al. (2010), ties the issue together by examining the contributions of psychosocial dynamics to health and quality of life and arguing for the importance of an integrated biopsychosocial approach to the study of longevity and centenarians. The authors highlight recent data to demonstrate the impact of four pertinent psychosocial domains for future longevity research: (1) demographics, life events, and personal history; (2) personality; (3) cognition; (4) socioeconomic resources and support systems. L. W. Poon et al. (2010) recommend that the above items supplement the 2001 *NIA Panel on the Characterization of Participants in Studies of Exceptional Survival in Humans* [41] that was originally developed to provide guidelines on measures that are important for studies of exceptional survival.

This fine collection of manuscripts will no doubt add important insights to the growing knowledge base in the field of gerontology. As more and more of the global population joins *Generation “C”,* understanding what happens on the right tail of the survival curve will be critical for improving the quality of life of these special elders and, indeed, should help all of our elderly population. Centenarian studies have and will continue to be important contributors to the research agenda in aging and will no doubt yield more key discoveries in the quest for healthier and longer lives for us all.

*Donald Craig Willcox* *Bradley J. Willcox* *Leonard W. Poon*
